# Ferroptosis and Triple-Negative Breast Cancer: A Systematic Overview of Prognostic Insights and Therapeutic Potential

**DOI:** 10.7759/cureus.51719

**Published:** 2024-01-05

**Authors:** Mohsin Khan, Vyshnavidevi Sunkara, Mansi Yadav, Syed Faqeer Hussain Bokhari, Abdur Rehman, Azka Maheen, Abdullah Shehryar, Srikar P Chilla, Maheen Nasir, Humaira Niaz, Jinal Choudhari, Nabila N Anika, Maaz Amir

**Affiliations:** 1 Interventional Radiology, Musgrove Park Hospital, Taunton, GBR; 2 Internal Medicine, Katuri Medical College, Guntur, IND; 3 Internal Medicine, Pandit Bhagwat Dayal Sharma Post Graduate Institute of Medical Sciences, Rohtak, IND; 4 Surgery, King Edward Medical University, Lahore, PAK; 5 Surgery, Mayo Hospital, Lahore, PAK; 6 Medicine, Gomal Medical College, Dera Ismail Khan, PAK; 7 Internal Medicine, Allama Iqbal Medical College, Lahore, PAK; 8 Medicine, Care Hospitals, Hyderabad, IND; 9 School of Health Sciences, University of East London, London, GBR; 10 Anesthesiology, National University of Medical Sciences, Lahore, PAK; 11 Internal Medicine, Peshawar Medical College, Peshawar, PAK; 12 Research & Academic Affairs, Larkin Community Hospital, Miami, USA; 13 Medicine, Holy Family Red Crescent Medical College and Hospital, Dhaka, BGD; 14 Internal Medicine, King Edward Medical University, Lahore, PAK

**Keywords:** heterogeneity, therapeutic targets, biomarkers, systematic review, triple-negative breast cancer, prognosis, ferroptosis

## Abstract

In the realm of oncology, the prognosis and treatment of triple-negative breast cancer (TNBC) have long been challenges for researchers and clinicians. Characterized by its aggressive nature and limited therapeutic options, TNBC demands innovative approaches to understanding its underlying mechanisms and improving patient outcomes. One such avenue of exploration that has emerged in recent years is the study of ferroptosis, a form of regulated cell death driven by iron-dependent lipid peroxidation. Ferroptosis has garnered increasing attention due to its potential relevance in the context of TNBC. This systematic review aims to shed light on the intricate interplay between ferroptosis and the prognosis of TNBC. The article delves into a comprehensive examination of the existing literature to provide a holistic understanding of the subject. By investigating ferroptosis as both an intervention and a prognostic factor in TNBC, this article seeks to unravel its potential as a therapeutic target and prognostic marker. The emerging evidence and heterogeneity of ferroptosis in TNBC underscore the need for a systematic approach to assess its impact on patient outcomes. This review will serve as a valuable resource for researchers, clinicians, and healthcare professionals striving to enhance our knowledge of TNBC and explore novel avenues for prognosis and treatment.

## Introduction and background

Triple-negative breast cancer (TNBC) represents a highly aggressive and complex subtype of breast cancer characterized by the absence of three important receptors: the estrogen receptor (ER), the progesterone receptor (PR), and the human epidermal growth factor receptor 2 (HER2) [1]. This unique molecular profile distinguishes TNBC from other breast cancer subtypes, rendering it a challenging entity to manage. TNBC is associated with a more aggressive clinical course, earlier onset, and a higher probability of recurrence [2]. It typically affects younger women and has limited targeted treatment options due to the absence of the mentioned receptors [3]. One of the most pressing challenges in the realm of TNBC is its unpredictability and often unfavorable prognosis. The inherent heterogeneity of TNBC results in a wide spectrum of clinical outcomes, ranging from early-stage curable disease to rapidly progressing metastatic conditions. This variability in patient response to treatment and survival outcomes has been the subject of intense research aimed at unraveling the underlying factors that drive these disparities.

Ferroptosis is a form of regulated cell death that has garnered significant attention in recent years, especially in the context of cancer research. It is characterized by the accumulation of lipid peroxides and is distinct from other forms of cell death such as apoptosis and necrosis [4]. Ferroptosis exerts a pivotal role in cellular homeostasis, primarily through the regulation of iron-dependent lipid peroxidation processes. This unique cell death pathway is particularly intriguing due to its potential to impact cancer biology and treatment outcomes [5]. The rationale for exploring ferroptosis in the context of TNBC stems from several compelling factors. First, TNBC's aggressive nature and limited treatment options necessitate the exploration of novel therapeutic avenues. Recent studies have indicated that TNBC may exhibit differential sensitivity to ferroptosis compared to other breast cancer subtypes [6-8]. Understanding the interplay between ferroptosis and TNBC can provide critical insights into the disease's pathogenesis and offer innovative treatment strategies.

This systematic review aims to address the fundamental question: What is the relationship between ferroptosis and the prognosis of triple-negative breast cancer (TNBC)? We will investigate the mechanisms through which ferroptosis influences TNBC outcomes, the identification of potential biomarkers and therapeutic targets that may enhance prognosis, and the impact of ferroptosis heterogeneity on patient responses to treatment. The significance of this systematic review lies in its potential to bridge the existing knowledge gap between ferroptosis and TNBC prognosis. By providing a comprehensive overview of the current literature and conducting a critical analysis of the data, this review will shed light on the intricate relationship between ferroptosis and TNBC, ultimately guiding future research endeavors and the development of targeted therapeutic interventions. Understanding how ferroptosis impacts TNBC prognosis has the potential to revolutionize the management of this aggressive breast cancer subtype and improve patient outcomes.

In the subsequent sections of this systematic review, we will delve into the existing body of literature, dissecting the role of ferroptosis in TNBC, the identification of biomarkers, potential therapeutic targets, and the implications of ferroptosis heterogeneity in shaping the prognosis of this challenging malignancy.

## Review

Materials and methods

Search Strategy

In our pursuit to investigate the intricate relationship between ferroptosis and the prognosis of TNBC, we carefully designed a thorough search strategy following PRISMA guidelines. This strategy involved several key steps to ensure the comprehensive coverage of relevant scientific literature. To start, we conducted a detailed search across reputable databases, including PubMed, Embase, Web of Science, and Scopus, which acted as our primary sources of scientific information. To refine our focus, we thoughtfully crafted a set of specific keywords, such as "Ferroptosis," "Triple-negative breast cancer," "Prognosis," "Biomarkers," "Treatment," and others, to guide our search and explore all relevant aspects of the topic. We utilized Boolean operators like "AND" and "OR" to combine these keywords in various combinations, helping us discover a wide range of pertinent studies. For instance, the query "ferroptosis AND triple-negative breast cancer AND prognosis" was particularly instrumental in narrowing down our research interests. The time frame for our search was from inception to November 2023, ensuring that we included all the relevant research while maintaining thoroughness. With this well-crafted strategy, we successfully identified and analyzed numerous relevant studies, forming the foundation of our systematic review on ferroptosis and its impact on the prognosis of TNBC.

Eligibility Criteria

In establishing the eligibility criteria for this systematic review, a meticulous approach has been undertaken to ensure the utmost rigor and relevance. The inclusion criteria encompass several key dimensions. Firstly, the study type under consideration includes peer-reviewed research articles, cohort studies, and clinical trials. This broad spectrum ensures the incorporation of high-quality, evidence-based research, crucial for a comprehensive review. The population of interest is limited to patients diagnosed with TNBC, aligning the research focus with the specific population under investigation. Studies investigating ferroptosis as an intervention or exploring its role in the prognosis of TNBC are included, as these directly address the central research inquiry. Additionally, studies reporting on prognostic factors, survival rates, or clinical outcomes associated with ferroptosis in the context of TNBC are considered, providing valuable insights into the impact of ferroptosis on patient outcomes. To maintain linguistic consistency, only studies published in the English language are incorporated, given its prominent role as the primary language of communication in the scientific community.

Conversely, the exclusion criteria are designed to uphold the precision and integrity of this review. Irrelevant studies that do not pertain to the relationship between ferroptosis and the prognosis of TNBC are excluded to maintain focus and relevance. Furthermore, studies conducted solely on animal models are omitted, as the primary objective is to concentrate on human-relevant research. Non-English language publications are excluded due to language limitations, ensuring a comprehensive understanding and interpretation of the research. Conference abstracts, posters, and unpublished works fall under the category of gray literature and are excluded to maintain the quality and reliability of the included studies. Studies with insufficient data to adequately address the research question are excluded to maintain the quality and reliability of the included research.

Data Extraction

The data extraction for this systematic review followed a rigorous and systematic approach to ensure the reliability and validity of the findings. The process can be divided into two stages. In the first stage, articles were screened based on their title and abstracts. The selected titles and their corresponding abstracts were reviewed to gain a preliminary understanding of their relevance to the research topic. Two independent reviewers meticulously assessed the abstracts for relevance. The assessment categorized each article as "relevant," "not relevant," or "probably relevant" based on a careful evaluation.

The second stage involved a detailed examination of the full-text articles for eligibility. Two independent reviewers were responsible for extracting data from the selected source publications. This data extraction was conducted using a standardized Microsoft Excel data extraction form (Microsoft Corporation, Redmond, WA, USA). Each reviewer independently applied the predefined inclusion and exclusion criteria to potentially eligible studies. In the event of any discrepancies or disagreements between the two primary reviewers, a third reviewer was involved to independently review and settle the differences through discussion. This consensus-seeking process ensured the accuracy and consistency of the data extraction and eligibility assessment.

The data extraction form gathered essential information from the selected studies, including details about the research's authors, publication year, country of origin, participant characteristics, study settings, study design, outcome measures utilized, and major results. This comprehensive data collection enabled a detailed analysis of the study's findings and ensured that all pertinent information was considered.

Data Analysis and Synthesis

The authors analyzed and synthesized the results using a narrative text approach focused on the relationship between ferroptosis and the prognosis of TNBC.

Results

Study Selection Process

**Figure 1:**
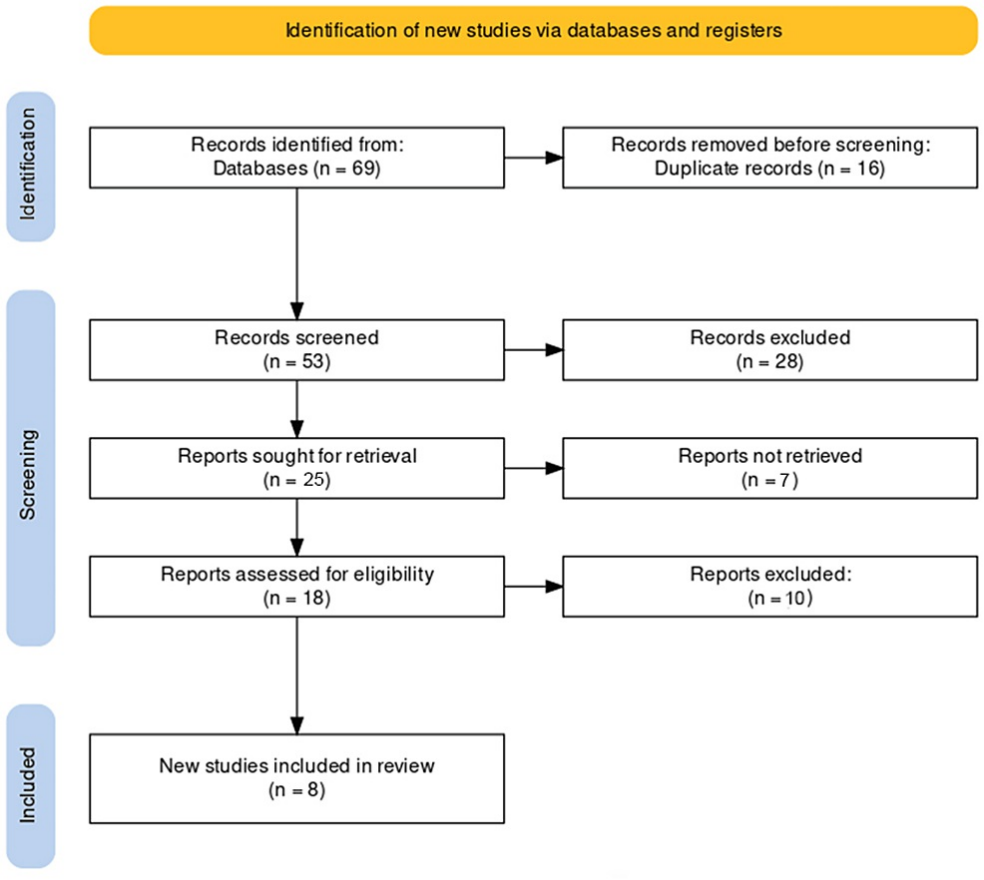
PRISMA flow diagram of the selection of studies for inclusion in the systematic review PRISMA: Preferred Reporting Items for Systematic Reviews and Meta-Analyses

The examination of four database searches resulted in the identification of 69 articles. Following the removal of 16 duplicates, the titles and abstracts of the remaining 53 publications were assessed. Subsequently, 24 potential studies were identified, and their eligibility was verified by examining the full texts. Ultimately, eight articles met the inclusion criteria. No studies that fulfilled the eligibility criteria were encountered while scrutinizing the references of the selected articles. A visual representation of this process is illustrated in the Preferred Reporting Items for Systematic Reviews and Meta-Analyses (PRISMA) flowchart (Figure 1).

Characteristics of Selected Studies

Overall, eight papers met the inclusion criteria. Four studies were in vitro cell culture studies, three were cohorts, and one was mixed (both in vitro cell culture and cohort). Seven studies were conducted in China, and one was in Italy. The main findings and characteristics of the included studies are mentioned in Table 1.

**Table 1:** A summary of the studies included in this systematic review

Author	Year	Country	Study Type	Cell Lines or Genes Studied	Instruments	Main Findings
Consoli et al. [9]	2022	Italy	In vitro Cell Culture	Human breast cancer cell lines: MCF-7 and MDA-MB 231	Cell culture, MTT assay, qRT-PCR, Western blot analysis, Measurement of HO enzymatic activity, ELISA, Measurement of intracellular Fe2+ content, Measurement of lipid peroxidation, Measurement of mitochondrial membrane potential, Determination of thiol groups, Measurement of ROS levels.	The study aimed to investigate the role of heme oxygenase (HO) in ferroptosis induction in breast cancer cell lines, focusing on MDA-MB 231 cells. The results revealed that MDA-MB 231 cells were more sensitive to ferroptosis compared to MCF-7 cells, with erastin, a ferroptosis inducer, significantly reducing their viability. Erastin upregulated HO-1 expression and activity, while hemin, an HO substrate, accelerated ferroptotic cell death, increasing ROS and altering the cellular redox balance. Silencing HO-1 reduced erastin's cytotoxic effect, and curcumin, another HO-1 inducer, induced features of ferroptosis, including increased ROS and lipid peroxidation. The study provides insights into the role of HO-1 in regulating ferroptosis in breast cancer cells, offering potential implications for cancer therapy.
Ding et al. [10]	2021	China	In vitro Cell Culture	Human triple-negative breast cancer cell lines: MDA-MB-231, SUM159, BT574, Hs578T, and MDA-MB-468	Cell viability assays (MTT, AlamarBlue, ATP), flow cytometry, Western blotting, real-time PCR for gene expression analysis, immunofluorescence, immunohistochemistry, high-content screening, animal studies, microscopy (fluorescence), and Liquid Chromatography-Mass Spectrometry for drug concentration analysis	The study investigated the small molecule DMOCPTL's impact on TNBC by inducing ferroptosis and apoptosis. DMOCPTL exhibited potent anti-proliferative effects on TNBC cells by directly interacting with and ubiquitinating GPX4, a protein involved in protecting cells from ferroptosis. This process led to GPX4 degradation, promoting ferroptosis and apoptosis. The study also highlighted the role of EGR1 in inhibiting apoptosis in TNBC cells. Furthermore, the prodrug Compound 13 derived from DMOCPTL demonstrated efficacy in inhibiting TNBC tumor growth in vivo without obvious toxicity. These findings suggest that DMOCPTL, by targeting GPX4 and EGR1, could be a promising therapeutic candidate for TNBC, offering potential improvements in TNBC patient prognosis.
Li et al. [11]	2022	China	In vitro Cell Culture	TNBC cell lines (MDA-MB-231, MDA-MB-436, HCC38, Hs578T) Luminal cell lines (MCF-7, ZR75-1, T47-D) HER2-enriched cell line SKBR3 Human embryonic kidney (HEK) 293 T cells	Cell viability and colony formation assays, synergy index (SI) analysis, drug screening, drug synergy assays, cell cycle assay, lipid peroxidation measurement, GSH measurements, ROS measurements, evaluation of malondialdehyde (MDA) and 4-Hydroxynonenal (4-HNE) levels, generation of drug-tolerant cells, RNA isolation and quantitative real-time PCR, animal models for tumor growth, RNA-Seq, Kyoto Encyclopedia of Genes and Genomes (KEGG) enrichment analysis, fatty acid analyses, and immunohistochemistry	Combining CB1 antagonists with ferroptosis inducers like erastin and RSL3 displayed potent inhibition of TNBC cell growth, particularly with rimonabant. TNBC cells exhibited heightened sensitivity to this combination, emphasizing its unique effectiveness. The treatment triggered ferroptosis and cell cycle arrest, with elevated lipid peroxidation, malondialdehyde (MDA), and reactive oxygen species (ROS) production. CB1's role in regulating ferroptosis sensitivity was highlighted, with CB1 expression influencing TNBC cell responses to inducers. Moreover, key fatty acid metabolism enzymes, SCD1 and FADS2, were implicated in CB1-mediated ferroptosis sensitivity. CB1 activation engaged the PI3K-AKT and MAPK pathways, contributing to TNBC cell resistance, partially reversible by inhibiting these pathways. In a mouse model, the combination therapy significantly reduced TNBC tumor growth, suggesting clinical potential. These findings propose a promising therapeutic strategy for TNBC, offering hope for improved patient outcomes.
Wu et al. [12]	2022	China	Cohort	Ferroptosis related genes: IFNG, GABARAPL1, FH, BRD4, TFAP2C, MT1G, WIPI1, FADS2, SLC2A12, NRAS, DUOX1, HSF1, CISD1, SLC1A5, SLC2A8	Bioinformatics analysis, establishment of a prognostic prediction model, quantitative real-time polymerase chain reaction (qRT-PCR), and functional enrichment analysis	The findings of this study related to the prognosis of TNBC are significant. The researchers established a prognostic model based on 15 ferroptosis-related genes, allowing them to classify TNBC patients into high-risk and low-risk groups. Patients in the high-risk group exhibited notably worse survival outcomes than those in the low-risk group, as demonstrated by Kaplan-Meier analysis and high accuracy in time-dependent receiver operating characteristic (ROC) curve analysis. This indicates that the expression patterns of these ferroptosis-related genes can serve as robust predictors of TNBC prognosis, offering valuable insights into identifying patients at higher risk who may require more aggressive treatment strategies. The study highlights the potential clinical significance of ferroptosis-related genes in improving the management and prognosis of TNBC, which has a historically poor outcome among breast cancer subtypes.
Sun et al. [13]	2022	China	In vitro Cell Culture	TNBC cell line: MDA-MB-231	CCK-8 assay, Transmission Electron Microscopy (TEM), Flow Cytometry, Laser Scanning Confocal Microscopy	The study observed the effects of propofol, PIE, and fospropofol disodium on MDA-MB-231 cells, focusing on their proliferation, apoptosis, intracellular ROS levels, and potential involvement in ferroptosis. The results indicate a significant dose-dependent decrease in cell proliferation with propofol treatment. Combinations of propofol/PIE/fospropofol disodium with doxorubicin or paclitaxel showed synergistic inhibition of proliferation compared to chemotherapy alone. The analysis of apoptosis revealed morphological changes indicative of apoptotic processes in cells treated with these agents. The study also found an increase in intracellular ROS levels in cells treated with propofol/PIE, both alone and in combination with doxorubicin or paclitaxel. Propofol and PIE, in particular, demonstrated the potential to induce ferroptosis-related morphological changes in mitochondria. The study suggests the involvement of the p53-SLC7A11-GPX4 pathway in propofol-induced ferroptosis.
Yu et al. [14]	2019	China	In vitro Cell Culture	Human TNBC cell line (MDA-MB-231) Human fetal lung fibroblasts (HFL-1 )	High-Performance Liquid Chromatography (HPLC), Fluorescence microscopy, Western blot, MTT assay, JC-1 Apoptosis Detection Kit, Flow cytometry	The study found that targeted exosome-encapsulated erastin effectively induced ferroptosis in TNBC cells (MDA-MB-231). The incorporation of erastin into exosomes enhanced its delivery and uptake by the cancer cells, leading to increased cell death. These findings suggest that this targeted approach could improve the prognosis of TNBC by providing a more effective method for inducing cancer cell death while potentially minimizing damage to healthy cells.
Yuan et al. [15]	2023	China	Cohort, In vitro Cell Culture	Main gene: STEAP3 TNBC cell lines: (MDA-MB-231, MDA-MB-468, BT-549) Non-TNBC cell lines: (MCF-7, T-47D, BT-474)	Differential expression analysis, survival analysis, nomogram development and validation, prognostic signature model, cell culture, RT-qPCR, Western immunoblotting, and immunohistochemical staining	This study identified a set of ferroptosis-related genes (FRGs) in triple-negative breast cancer (TNBC) and found that 87 of these genes were differentially expressed in TNBC tumors. Among the differentially expressed FRGs, two genes, CISD1 and STEAP3, were associated with a higher risk and poorer overall survival in TNBC patients. A prognostic nomogram and a prognostic DE-FRG signature model were developed to predict TNBC patient outcomes. Importantly, the study highlighted the specific role of STEAP3, which was upregulated in TNBC and linked to worse patient prognosis. Additionally, TNBC patients with higher STEAP3 expression were found to be more sensitive to certain chemotherapeutic drugs, suggesting potential treatment implications. These findings shed light on the potential significance of ferroptosis-related genes, particularly STEAP3, in the prognosis and treatment of TNBC.
Zhang et al. [16]	2023	China	In vitro Cell Culture	Human breast cancer cell lines (BT474, MDA-MB-468, MCF7, SKBR3, and MDA-MB-231) Human normal breast cell line (MCF10A)	qRT-PCR, Western blot assays, flow cytometry, and bioinformatics tools for analyses such as Kaplan-Meier curves, genetic alteration analysis, and functional enrichment analysis	The main findings indicate that higher MTHFD2 expression is associated with a poorer prognosis in breast cancer, specifically in TNBC. The study also explores the potential link between MTHFD2 and ferroptosis, suggesting that MTHFD2 may play a role in regulating this process in TNBC cells. This finding opens avenues for further understanding the molecular mechanisms underlying TNBC prognosis.

Discussion

The exploration of ferroptosis in the context of triple-negative breast cancer (TNBC) presents a compelling avenue for understanding the underlying mechanisms influencing prognosis and treatment outcomes. This systematic review delves into the intricate relationship between ferroptosis and TNBC, shedding light on its potential as both a prognostic marker and a therapeutic target.

The findings from the reviewed literature suggest a multifaceted interplay between ferroptosis and TNBC prognosis. Ferroptosis, characterized by iron-dependent lipid peroxidation, plays a pivotal role in cellular homeostasis, and its dysregulation has been implicated in various cancers, including TNBC. Several studies underscore the importance of exploring the molecular pathways through which ferroptosis influences TNBC outcomes. For instance, the downregulation of key regulators of ferroptosis, such as glutathione peroxidase 4 (GPX4), has been associated with increased lipid peroxidation and enhanced TNBC cell death. Moreover, the intricate crosstalk between ferroptosis and other cellular processes, such as autophagy and oxidative stress, adds complexity to the understanding of TNBC progression [17,18].

Identification of reliable biomarkers and therapeutic targets is crucial for advancing the clinical implications of ferroptosis in TNBC. The literature highlights potential biomarkers associated with ferroptosis sensitivity or resistance in TNBC. For instance, high levels of lipid peroxidation markers, such as malondialdehyde (MDA) and 4-Hydroxynonenal (4-HNE), have been correlated with ferroptosis induction in TNBC cells [19]. Additionally, exploring the role of specific proteins, including system xc- and acyl-CoA synthetase long-chain family member 4 (ACSL4), as potential therapeutic targets has garnered attention. The modulation of these markers and targets may offer a promising approach to enhancing TNBC prognosis by manipulating ferroptosis [20].

The heterogeneous nature of ferroptosis responses in TNBC adds a layer of complexity to its prognostic implications. Variability in ferroptosis sensitivity among different TNBC subtypes and individual patients underscores the need for personalized therapeutic approaches. Understanding the factors contributing to this heterogeneity, such as genetic mutations and microenvironmental influences, is paramount for tailoring treatment strategies. This review emphasizes the importance of considering ferroptosis as a dynamic process with diverse implications for TNBC prognosis [21,22].

The integration of ferroptosis into the clinical management of TNBC holds great promise. The reviewed literature suggests that targeting ferroptosis-related pathways may provide a novel therapeutic avenue for overcoming the limitations of current treatments. Clinical trials investigating ferroptosis modulators in TNBC are underway, emphasizing the translation of preclinical findings into actionable strategies. As we move forward, understanding the dynamic interplay between ferroptosis and other treatment modalities, such as chemotherapy and immunotherapy, will be crucial for optimizing combination therapies and improving overall patient outcomes [23,24].

## Conclusions

This systematic review investigates the interplay between ferroptosis and the prognosis of triple-negative breast cancer (TNBC), a challenging and aggressive subtype with limited treatment options. Ferroptosis, a form of regulated cell death driven by iron-dependent lipid peroxidation, emerges as a focal point due to its potential therapeutic relevance. The review, encompassing eight studies, reveals the heterogeneity of ferroptosis responses in TNBC and emphasizes the need for a systematic approach to assess its impact on patient outcomes. The selected studies, ranging from in vitro cell culture to cohort analyses, explore various aspects, including the role of specific genes, targeted therapies, and the development of prognostic models based on ferroptosis-related genes. Findings suggest that ferroptosis-related genes and biomarkers hold promise as prognostic indicators, opening avenues for personalized treatment strategies. As clinical trials investigating ferroptosis modulators in TNBC progress, the integration of ferroptosis into clinical management offers a paradigm shift, emphasizing optimized combination therapies and improved patient outcomes. Overall, this article provides a comprehensive overview, serving as a valuable resource for researchers and clinicians navigating the complex landscape of TNBC and offering insights into potential therapeutic targets and prognostic markers.
